# A phosphite-based screening platform for identification of enzymes favoring nonnatural cofactors

**DOI:** 10.1038/s41598-022-16599-0

**Published:** 2022-07-21

**Authors:** Yuxue Liu, Zhuoya Li, Xiaojia Guo, Xueying Wang, Zongbao K. Zhao

**Affiliations:** 1 Henan Engineering Laboratory for Bioconversion Technology of Functional Microbes, College of Life Science, Henan Normal University, Xinxiang, 453007 China; 2 Division of Biotechnology, Dalian Institute of Chemical Physics, Chinese Academy of Sciences, Dalian, 116023 China

**Keywords:** Biochemistry, Biological techniques

## Abstract

Enzymes with dedicated cofactor preference are essential for advanced biocatalysis and biomanufacturing, especially when employing nonnatural nicotinamide cofactors in redox reactions. However, directed evolution of an enzyme to switch its cofactor preference is often hindered by the lack of efficient and affordable method for screening as the cofactor per se or the substrate can be prohibitively expensive. Here, we developed a growth-based selection platform to identify nonnatural cofactor-dependent oxidoreductase mutants. The growth of bacteria depended on the nicotinamide cytosine dinucleotide (NCD) mediated conversion of non-metabolizable phosphite into phosphate. The strain BW14329 lacking the ability to oxidize phosphite was suitable as host, and NCD-dependent phosphite dehydrogenase (Pdh*) is essential to the selection platform. Previously confirmed NCD synthetase with NCD synthesis capacity and NCD-dependent malic enzyme were successfully identified by using the platform. The feasibility of this strategy was successfully demonstrated using derived NCD-active malic enzyme as well as for the directed evolution of NCD synthetase in *Escherichia coli*. A phosphite-based screening platform was built for identification of enzymes favoring nonnatural cofactor NCD. In the future, once Pdh variants favoring other biomimetic or nonnatural cofactors are available this selection platform may be readily redesigned to attain new enzyme variants with anticipated cofactor preference, providing opportunities to further expand the chemical space of redox cofactors in chemical biology and synthetic biology.

## Introduction

**Figure 1:**
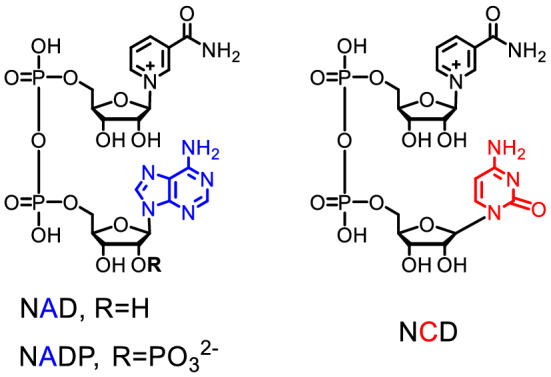
Chemical structures of NAD(P) and NCD.

The natural nicotinamide-based cofactors, nicotinamide adenosine dinucleotide (NAD, Fig. 1), nicotinamide adenine dinucleotide phosphate (NADP), and their reduced forms NAD(P)H are indispensable cofactors in biomanufacturing. Recent research highlights the value of nonnatural nicotinamide cofactors (mNADs), such as nicotinamide cytosine dinucleotide (NCD, Fig. 1), nicotinamide flucytosine dinucleotide, 1-benzyl-1,4-dihydropyridine-3-carboxamide, amino acid-based NAD analogs, 4′-thioribose NAD, 1-phenylnicotinamide, as well as nicotinamide mononucleotide (NMN), an endogenous metabolite^1–6^. These mimics have been explored and synthesized as alternatives to NAD(P) during biotransformation as mNADs are often inexpensive and bioorthogonal to the extraordinary complex redox systems^2,7^. The majority of mNADs may greatly reduce the cost of biotransformation, and has led to a major breakthrough both in in vitro biocatalysis and in vivo metabolic engineering^8,9^. So far, the mNADs-derived biotransformation processes involve oxidoreductase enzymes ^9,10^. To facilitate mNADs-dependent biotransformations, it is essential to engineer enzymes to favor mNADs, and various studies have focused on optimizing cofactor preference of enzyme^11^.

NCD is the only artificial coenzyme which was successfully biosynthesized and used in orthogonal redox reactions intracellularly, as demonstrated in our earlier studies^12–14^. Various NCD-favoring oxidoreductase, such as malic enzyme, phosphite dehydrogenase, d-lactate dehydrogenase, formate dehydrogenase, and formaldehyde dehydrogenase, have been successfully designed^2,15–18^. However, these enzymes are not enough for the wide application of NCD in the future. Thus, one of the challenges remains the efficient directed evolution of NCD-dependent enzymes. Although a colorimetric method is useful in screening NCD-favoring mutants, it is labor-intensive.

High throughput screening method that can correctly identify rare positive hits from diverse mutant libraries is critical to directed evolution. However, a successful directed evolution is often hindered by the lack of efficient and affordable selection methods, especially involving enormous mutant libraries^19^. Various methods have been developed to identify mNADs-active dehydrogenase mutants depending on mass spectrometry and absorbance spectroscopic change^2,3,20,21^. However, these methods are labor-intensive and time-consuming without the robotic systems, and result in a low throughput^22^. By contrast, the growth complementation method, which couples the examined enzyme property with the fitness of the host cell, is not dependent on intensive labor^23^. This approach has been successful developed and used to evolve NAD(P)H-dependent oxidoreductases based on redox balance principles in engineered *Escherichia coli* (*E. coli*) strains with disrupted intracellular cofactor cycling^24,25^. A recent growth-based selection strategy has since been applied in engineering NMN-dependent enzymes by linking *E. coli* growth to the NMN cycle with OD_600nm_ as the readout^4^. Therefore, we hypothesized that if NCD balance can be linked to *E. coli* growth, the mentioned selection strategy might be adapted to evolve NCD-active enzymes.

Here, we built a phosphite-based selection platform for the initial screening of the libraries to identify NCD-active mutant. Growth was utilized as the readout in the selection platform. The selection platform operates based on NCD-drive phosphite metabolism. Briefly, phosphite serves as the sole phosphorous source for *E. coli*. When the native phosphite oxidation pathway is disrupted, cell growth would rely on the heterologously introduced NCD redox cycle. NCD-drive phosphite oxidation reaction is catalyzed by NCD-dependent phosphite dehydrogenase (Pdh_I151R/P176R/M207A, Pdh*), a mutant with robust NCD preference^15^. NCD synthetase, which was created in our previous study, is employed for the in vivo NCD biosynthesis from CTP and NMN^22^. Then, a closed NCD redox cycle will be formed with the NCD-dependent oxidoreductase mutant to regenerate NCD. The feasibility of this strategy was proved using NCD-dependent malic enzyme (ME-L310R/Q401C, ME*) as the candidate^2^. We hypothesized that if phosphite dehydrogenase can be engineered to favor other mNADs, such a paradigmatic selection scheme might be designed and applied in engineering diverse mNADs-favoring oxidoreductase, as well as mNADs synthetase in the future.

## Results and discussion

### Design of the phosphite-based selection system

**Figure 2:**
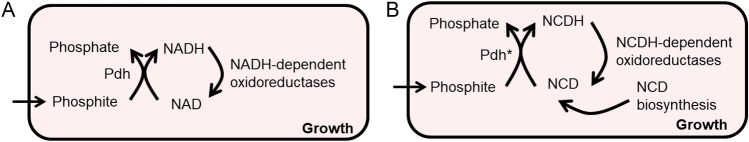
Schematics of phosphite-based selection system. (**A**) Phosphite was oxidized by Pdh and a closed redox loop was formed with NADH-dependent reactions to support growth of *E. coli* (*∆phn, ∆phoA*) with phosphite as the sole phosphorous source. (**B**) A closed NCD redox cycle promotes phosphite metabolism to support growth with phosphite as the sole phosphorous source. Phosphite cannot be oxidized in the absence of any of Pdh*, NCD, or NCDH-dependent oxidoreductase, and results in no growth of strain.

The design of our selection system relies on a closed NCD cycle to drive the oxidation of phosphite in *E. coli* (Fig. 2). It consists of four important and basic elements: engineered *E. coli* that cannot use phosphite as the sole phosphorous source, NCD, heterogenous NCD-depend phosphite oxidation pathway, and NCD regeneration pathway. The growth of *E. coli* was associated with the NCD cycle by the phosphite oxidation. This was achieved by disrupting endogenous phosphite metabolism and directing NCD-dependent phosphite dehydrogenase (Pdh*) into the life-essential phosphorus metabolism. Since cells cannot biosynthesize NCD autonomously, a NCD synthetase that created in our previous research by reprograming the *E. coli* nicotinic acid mononucleotide adenylyltransferase (NadD) to use CTP and NMN as substrates can be employed for the in vivo NCD biosynthesis^22^. In the presence of intracellular NCD and Pdh*, cell growth was restored only when a closed NCD redox cycle was formed with a NCD-active oxidoreductase. It suggested that this system can be used to screen for NCD-active oxidoreductase. Furthermore, this system also had the potential to screen NCD synthetase under the condition of complete NCD-cycle. In this system, the specific functions of NCD and the complementary enzymes are not linked to cell survival and they can be exchanged. Therefore, we anticipate that the phosphite-based selection will be highly instrumental in engineering diverse NCD-dependent oxidoreductases and NCD synthetase.

**Figure 3:**
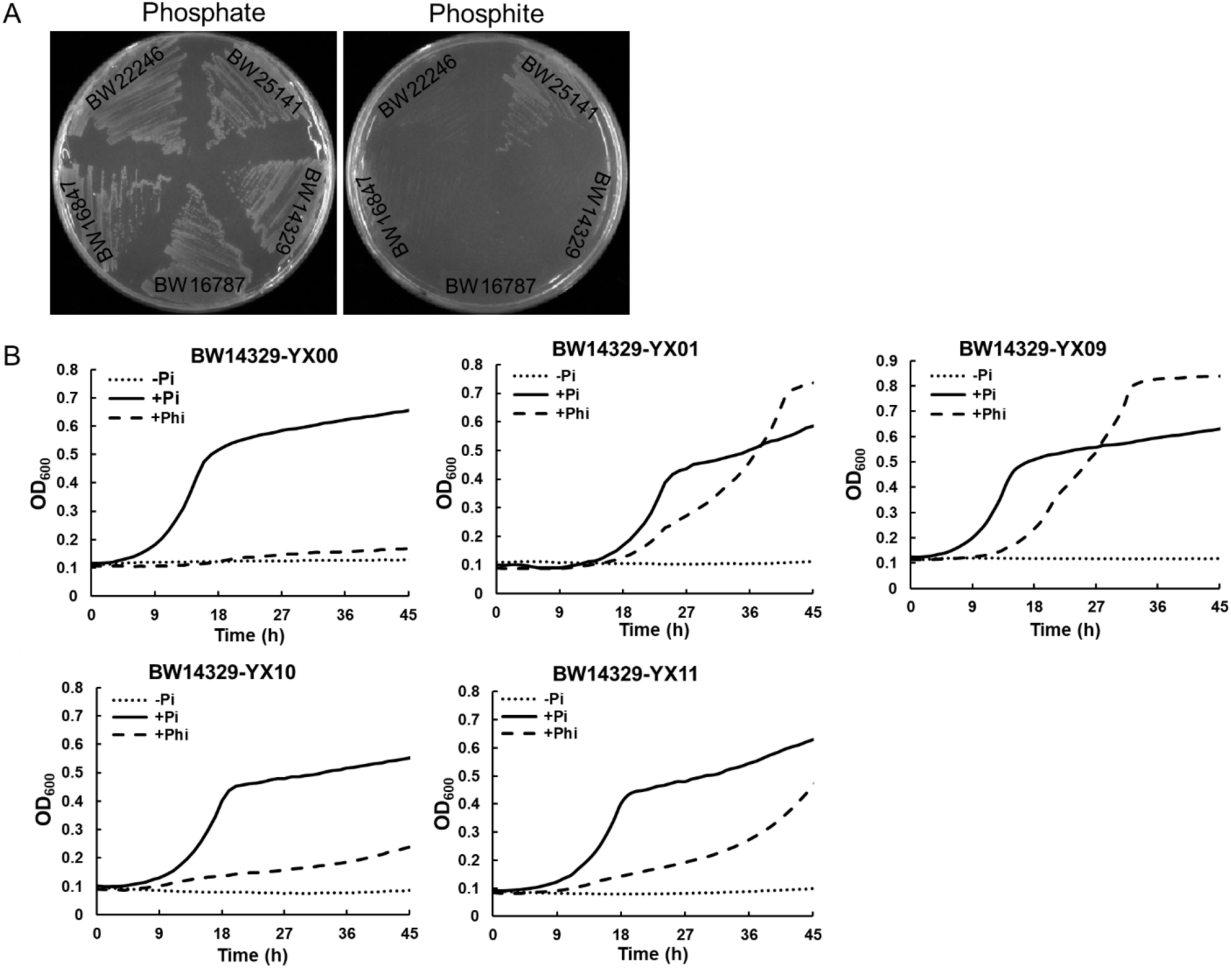
Growth of *E. coli* strains in MOPS minimal media supplemented with different phosphorous sources. (**A**) Growth behavior of strains cultured on solid medium. The concentration of phosphate or phosphite is 1 mM. (**B**) Growth behavior of engineered strains cultured in liquid medium supplemented with phosphate (solid line) or phosphite (dashed line) as phosphorous sources and without phosphorus sources (dotted line). Phi, phosphite. Pi, phosphate. BW14329-YX00, BW14329-YX01, BW14329-YX09, BW14329-YX10 and BW14329-YX11 were with no Pdh, wild type NAD-dependent Pdh, Pdh_I151R, Pdh_I151R/P176R/M207A and Pdh_I151R/P176E expression, respectively. All values reflect the average of three independent cultures.

As the selection system depending on phosphite metabolism to provide phosphorous source for cell growth, we first sought to select appropriate host that could not oxidize phosphite. *E. coli* is commonly used host strain for directed evolution of protein. However, *E. coli* has two independent pathways for oxidizing phosphite to phosphate depending on the *phn* operon and the *phoA* locus respectively^26,27^. Strains BW14329, BW16787, BW16847 and BW22246 were with the deletion of *phoA* and varying degrees of deletion of gene cluster *phn.* The capacity to oxidize phosphite of these strains were demonstrated by their ability to grow in MOPS minimal media with phosphite as the sole phosphorous source (Fig. 3A). All tested strains could grow normally on media with phosphate as phosphorous sources. It was consistent with expectations that, engineered strains with double knockout of *phoA* and *phn* cannot grow on phosphite medium compared with the control strain BW25141. Therefore, strains BW14329, BW16787, BW22246 and BW16847 can be used as host in the phosphite-based selection system. BW14329 was randomly selected as the host strain in the following study.

### Biorthogonality of phosphite dehydrogenase mutant

Based on the principle that the growth of the host cell depending on NCD-dependent phosphite metabolism, this selection system requires highly active and specific NCD-dependent phosphite dehydrogenase. NCD-dependent phosphite dehydrogenase could not provide phosphorus source for cell growth depending on NAD. Hence, we tested biorthogonality of Pdh* by monitoring the growth of engineered strains expressing different phosphite dehydrogenases. In our previous work, the cofactor preference of a series of *Ralstonia *sp. strain 4506 derived Pdh mutants, including Pdh_I151R, Pdh_I151R/P176E and Pdh* (Pdh_I151R/P176R/M207A), were characterized. According to kinetic constants of these mutants (Table S1), although the mutants Pdh_I151R and Pdh_I151R/P176E had higher NCD preference, but they still retained high activity against NAD^15^. The high *K*_m_ value (4.7 mM) and the low *k*_cat_/*K*_m_ value (0.045 mM^−1^ s^−1^) for NAD indicated that only Pdh* had the lowest activity with intracellular NAD and had the potential to exhibit bioorthogonality in vivo.

Then, engineered strains, including BW14329-YX00, BW14329-YX01, BW14329-YX09, BW14329-YX10, and BW14329-YX11 (Table S1), were constructed by transferring plasmids expressing no Pdh, wild-type (WT) Pdh, Pdh_I151R, Pdh_I151R/P176E and NCD-dependent Pdh* into the host strain BW14329, respectively. As expected, all engineered strains enabled growth in MOPS minimal media under 2 mM phosphate. When replaced with phosphite as the sole phosphorous source, engineered strains showed different growth states (Fig. 3B, Table 1). BW14329-YX01 and BW14329-YX09 can grow well at a fast specific-growth rate, 0.11 ± 0.00 h^−1^ and 0.12 ± 0.00 h^−1^ respectively. Due to relatively low NAD activity, BW14329-YX11 grew to a certain extent at a lower specific-growth rate (0.07 ± 0.00 h^−1^). Attributing to the high cofactor specificity of Pdh*, BW14329-YX10 grew at the lowest growth rate (0.03 ± 0.00 h^−1^) under 2 mM phosphite, and the growth was very weak compared to BW14329-YX01. Although the biorthogonality of Pdh* in vivo was not strictly, these results suggested that the reaction mediated by Pdh* could potentially be used for the growth-based selection for NCDH-consuming reactions of interest.

**Table 1:** Maximum specific growth rate (h^−1^) comparison between strains under different culture regime.

Culture regime	BW14329-YX00	BW14329-YX01	BW14329-YX09	BW14329-YX10	BW14329-YX11
Phosphate	0.13 ± 0	0.12 ± 0	0.13 ± 0	0.13 ± 0.01	0.11 ± 0
Phosphite	0.01 ± 0	0.11 ± 0	0.12 ± 0	0.03 ± 0	0.07 ± 0

All values reflect the average of three independent cultures.

### Application of the selection method in directed nicotinate-mononucleotide adenylyltransferase evolution

**Figure 4:**
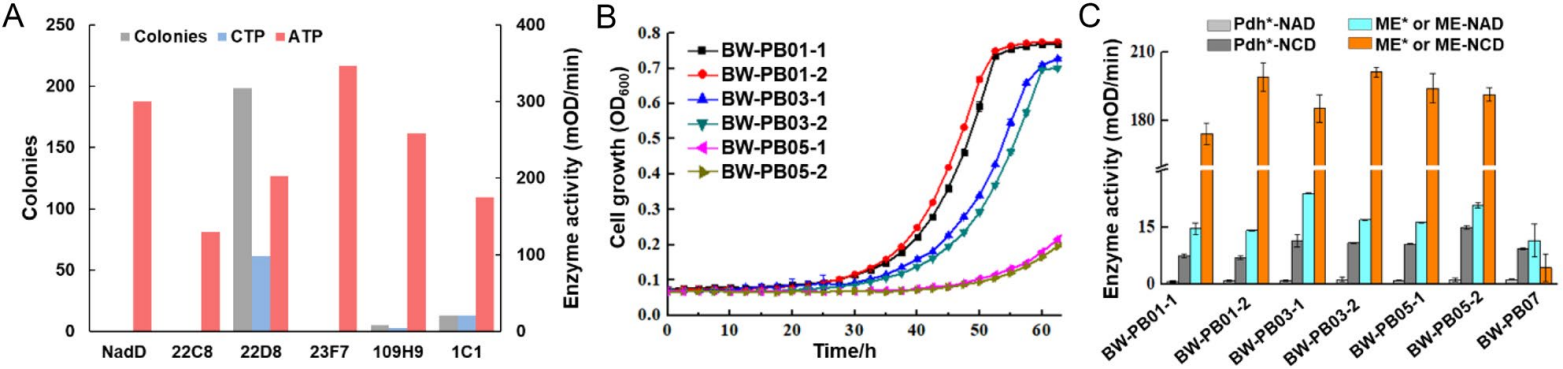
In vivo NCD synthesis supports *E. coli* growth. (**A**) Crude enzyme activities NadD mutants and number of colonies formed when transforming NadD variants. 22C8, 23F7, 109H9, 1C1, and 22D8 were NadD mutants formed during the directed evolution of NcdS. The data of crude enzyme activities was taken from the previous research^14^. (**B**) Growth characterization of engineered *E. coli* assembled with NCD-cycling pathway and NcdS in liquid minimal media with 5 mM phosphite as the sole phosphorous source. Data are the average of biological triplicates with standard deviations. (**C**) The crude enzyme activities of Pdh*, ME* and ME toward NAD and NCD. Experiments were conducted in triplicates, and data are presented as mean values. Pdh*-NAD, activity of Pdh* toward NAD. Pdh*-NCD, activity of Pdh* toward NCD. ME*or ME-NAD, activity of ME* or ME toward NAD. ME* or ME-NCD, activity of ME* or ME toward NCD. BW-PB01-1 and BW-PB01-2 had the same genotype, as well as BW-PB03-1 and BW-PB03-2, BW-PB05-1 and BW-PB05-2. BW-PB01, BW-PB03, and BW-PB05 expressed Pdh*, ME* and different NcdS. BW-PB07 expressed Pdh*, ME and NcdS2.

NadD catalyzes the synthesis of nicotinic acid adenine dinucleotide using ATP and nicotinic acid mononucleotide as substrates. NCD synthetase (NcdS) was created by reprograming the substrate-binding pockets of NadD, and catalyzed the condensation of CTP and NMN to form NCD^14^. As a proof-of-concept, we applied the selection method in directed evolution of NcdS. Cell growth will depend on the biosynthesis of NCD with presence of Pdh* and the NCD-cycle partner (Fig. 2B). The growth rate of strain will be positively correlated with the activity of NcdS. Here, we randomly selected WT NadD and several suspects formed during the directed evolution, including 22C8 (D22R), 23F7 (V23Q), 109H9 (D109R), 1C1 (P22K/C132L/W176L), and 22D8 (D22K), and the NCD synthesis capacity was enhanced sequentially according to previous report (Fig. 4A)^14^. The high capacity of NCD synthesis was reflected in high activity toward CTP and low activity toward ATP. The NCD cycle module was assembled on redesigned plasmid pUC18 (*bla::cat*) with Pdh* and ME* coexpression controlled under the *lac* and *ara* operon respectively, to give plasmid pUC-chl-(*P*_araB_)ME* + Pdh*. Plasmid expressing WT NadD or the variants and pUC-chl-(*P*_araB_)ME* + Pdh* were cotransformed into BW14329. We hypothesized that colonies only formed when NadD variants showed the high activity of NCD synthesis. In our results, colonies were only observed on MOPS plates with 5 mM phosphite when plasmid expressing 109H9, 1C1 or 22D8, but not WT NadD, 22C8 or 23F7 was transformed. Under the same conditions, colonies formed of 109H9, 1C1, and 22D8 were counted to be 5, 13, and 198, respectively. These results indicated that the number of colonies was positively correlated with the activity of mutant for NCD synthesis and this was in agreement with the screening principle.

To further prove our hypothesis, we tested the capacity of NcdS to regulate the growth of strains holding NCD-cycling pathway. We introduced NcdS-2, NcdS-3, and the V23Q/W176E mutant of NadD (3G8)^14^ on plasmid pUC-18 with Pdh* coexpression, giving pUC-chl-NcdS2 + Pdh*, pUC-chl-NcdS3 + Pdh*, and pUC-chl-3G8 + Pdh*. According to the previous research, NcdS-2 showed higher activity and preference of NCD biosynthesis than NcdS-3, whereas 3G8 had the lowest specificity^14^. The reconstructed plasmids were separately cotransformed with pTrc99K-ME* into BW14329, to give strains BW-PB01, BW-PB03, and BW-PB05, respectively. Growth behavior of engineered strains in MOPS media with phosphite as the sole phosphorous source was observed (Fig. 4B). Obviously, the higher activity of NcdS afforded an increased growth rate. It should be noted that the expression levels and activities of Pdh* and ME* influence the efficiency of phosphate production, which may affect the cell growth. In our results (Fig. 4C, Fig. S1), there was no significant difference in protein expression and crude enzyme activity for cofactors between different engineered strain. Therefore, the growth differences between strains were mainly caused by NcdS. These results suggested that the strain assembled with NCD-cycling pathway could potentially be used for the phosphite-based selection of NCD synthetase. Therefore, when NCD-cycling pathway was replaced by mNADs-cycling pathway, this system would potentially be applied to the selection of mNADs synthetase.

### Validation of screening system with evolved malic enzyme

**Figure 5:**
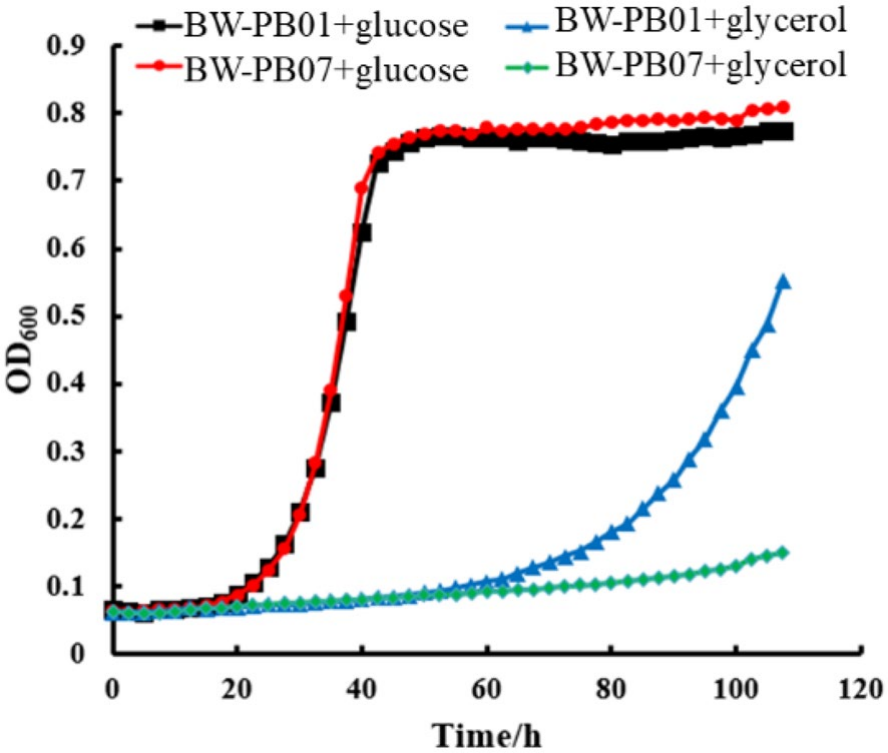
In vivo NCD cycling supports *E. coli* growth. Engineered strains assembled with different NCDH-consuming reactions were cultured in liquid minimal media with glucose or glycerol as carbon source and 5 mM phosphite as the sole phosphorous source. Data are the average of biological triplicates with standard deviations.

Figure 2B indicated that cell growth was restored only when the NCD cycle was closed. Hence, we tested if the NCD regeneration reaction could support growth with the presence of NCD and Pdh*. Plasmids pUC-NcdS-2 + Pdh* and pTrc99K-ME were cotransformed into BW14329 to give strain BW-PB07. Consistent with our expectations, when BW-PB01 was cultured in liquid minimal media with 0.4% glycerol and 5 mM phosphite, the NCDH-dependent ME* enabled growth with a long lag phase (Fig. 5). In contrast, BW-PB07 grew at a lower rate in the same condition when ME* was replaced by ME. However, the growth difference disappeared when glycerol was substituted for glucose. As the oxidation state of carbon sources has a significant effect on cellular NADH/NAD ratio, the intracellular NAD level was increased when glucose was used as carbon source compared to glycerol^28,29^. We speculate that the accumulated NAD may be consumed by the overexpressed Pdh* in the case of insufficient NCD, which promoted the phosphite metabolism and resulted in the growth of the cell. Overall, these results suggested that the selection platform could potentially be used for screening NCDH-consuming oxidoreductase.

## Conclusions

In summary, we have established a phosphite-based in vivo selection platform for NCDH-dependent reactions and NCD synthesis. ME and NCD-dependent mutant ME* were applied to test the selection system, and ME* was easy identified with the higher cell growth. On the other hand, we successfully applied the selection system toward to identify the variant with the higher activity that generated in the directed evolution of NcdS. Although the throughput and false positive rate were not demonstrated, this study suggested that NCDH-consuming enzymes can be identified by employing the in vivo selection process. It is not surprising that false hits would be identified from this phosphite-based screening platform. Further selection is needed to exclude false hits from the candidate by coupling this platform with a compatible colorimetric assay. As a result, the best performing enzyme variant would be identified. Despite the limitations, we envision that once Pdh variants favoring other biomimetic or nonnatural cofactors are available such a paradigmatic selection platform may be adaptable for mNADs-favoring enzymes and mNADs synthetase engineering in the future.

## Materials and methods

### Reagents and chemicals

DNA polymerase PrimeSTAR and restriction enzyme *Dpn*I were purchased from Takara. Isopropyl-β-d-thiogalactopyranoside (IPTG), l-arabinose (L-ara), ampicillin (Amp), chloramphenicol (Chl), and kanamycin (Kan) were purchased from Sangon Biothech (Shanghai, China). NCD was chemically synthesized as our previous procedure^2^. His-tag antibodies were purchased from Beyotime Biotechnology (Shanghai, China). Tanon™ ECL western blotting substrate kit was purchased from Tanon (Shanghai, China).

### Strains and plasmids

Bacterial strains and plasmids used in this study are listed in Tables S2 and S3, respectively. *E. coli* BL21 (DE3) was used for plasmid construction. *E. coli* BW14329, BW16787, BW22246 and BW16847 were obtained from the Coli Genetic Stock Center (CGSC). The construction of the plasmids is detailed in the “Genetic methods”.

### Media and growth conditions

Luria–Bertani broth was used for growth during cloning. MOPS minimal medium^30^ supplemented with different phosphorus sources was utilized for determining the growth behavior of the strains. Unless otherwise specified, MOPS medium was supplemented with 0.4 g/L glucose, and corresponding antibiotics (50 μg/mL Kan, 100 μg/mL Amp, 30 μg/mL Chl) and inducers (0.1 mM IPTG,1 mM L-ara). Seed cultures were grown in LB medium for protein induction at 25 °C at 200 rpm for 24 h, supplemented with 50 μg/mL Kan, 0.1 mM IPTG and 1 mM L-ara. Cells were collected, washed thrice and resuspended with 1 mL of MOPS medium without phosphorous source. A 3-μL volume of cell suspension was spotted on the corresponding gradient phosphite agar plate. The plate was cultured at 25 °C for 72 h. To determine the growth curve of the strains, the cell suspension was then inoculated into 200 μL of MOPS medium with an initial OD_600_ of 0.2 and cultivated at 25 °C with Bioscreen instrument. The absorbance at 600 nm was measured every 2 h. The specific-growth rate and lag-phase data were estimated from absorbance growth curves using the modified Gompertz model as described previously^31^. The method used to determine the activity of NadD variants by detecting the transformation efficiency was detailed in the Supporting Information.

### Genetic methods

Plasmids were constructed by restriction-free cloning^32^ and the plasmid pUC18 as the initial template (Fig. S2). Genes encoding ME and ME* were amplified from plasmids pTrc99K-ME and pTrc99K-ME*, which were lab collection. Genes encoding Pdh and Pdh* were amplified from plasmids pK-Pdh and pK-Pdh*, respectively. Pdh, ME, and NcdS were expressed with His×6 tag at the C-terminal.

### Western blot assay

Western blot was performed using His-tag antibodies to demonstrate the expression of Pdh*, ME/ME*, and NcdS in strains BW-PB01, BW-PB03, BW-PB05, and BW-PB07. About 5.0 × 10^9^ of cells were collected by centrifugation at 10,000×*g* at 4 °C for 5 min and washed twice with 1 mL of 10 mM Tris–Cl buffer (pH 8.0). The cells were resuspended in 200 μL of 10 mM Tris–Cl buffer. Cell pellets were disrupted by sonicator and the supernatant was achieved by centrifugation at 13,000×*g* for 10 min. Next, 5 μL of loading buffer was added to 15 μL of the supernatant and boiled for 10 min. Samples were subjected to SDS-PAGE and analyzed by western blot. Image data were obtained by Tanon™ ECL western blotting substrate kit and analyzed by Tanon-5200 Multi automatic chemiluminescence image processing system.

### Specific enzyme activity assays

To analyze crude enzyme activities of engineered, about 5.0 × 10^9^ of cells were treated by the above process. ME* and Pdh* activities were measured in a mixture containing 50 mM HEPES (pH 7.5), 0.05 mM NAD or NCD, 0.4 mM methylthiazolyldiphenyl-tetrazolium bromide, 1.0 mM phenazine ethosulfate, 10 mM MgCl_2_, and 5.0 mM l-malate for ME* or phosphite for Pdh*. The reaction was started by addition of 10 μL of enzyme solution. Reaction rate was determined by monitoring the increase of formazan in absorbance at 570 nm at room temperature with PowerWave XS universal microplate spectrophotometer.

## Supplementary Information

Supplementary Information 1.

Supplementary Information 2.

## Data Availability

All data generated or analyzed during this study are included in this article (and its Supplementary Information file).

## Abbreviations

NAD: Nicotinamide adenosine dinucleotide
NADH: Reduced form of nicotinamide adenosine dinucleotide
NCD: Nicotinamide cytosine dinucleotide
NCDH: Reduced form of nicotinamide cytosine dinucleotide
NADP: Nicotinamide adenine dinucleotide phosphate
NADPH: Reduced form of nicotinamide adenine dinucleotide phosphate
mNADs: Nonnatural nicotinamide cofactors
NMN: Nicotinamide mononucleotide
Pdh: Phosphite dehydrogenase
Pdh*: I151R/P176R/M207A mutant of phosphite dehydrogenase
ME: Malic enzyme
ME*: L310R/Q401C mutant of malic enzyme
NadD: Nicotinate-mononucleotide adenylyltransferase
NcdS: NCD synthetase
WT: Wild-type
IPTG: Isopropyl-β-d-thiogalactopyranoside
L-ara: L-arabinose
Amp: Ampicillin
Chl: Chloramphenicol
Kan: Kanamycin
