# Supplementary material for: A phosphite-based screening platform for identification of enzymes favoring nonnatural cofactors
Source: Sci Rep. 2022 Jul 21;12:12484. doi: 10.1038/s41598-022-16599-0 (PMC9304416; doi:10.1038/s41598-022-16599-0)
Supplement: Supplementary file 1 — Supplementary Information 1. [file 41598_2022_16599_MOESM1_ESM.docx]

**A Phosphite-Based Screening Platform for Identification of Enzymes Favoring Nonnatural Cofactors**

*Yuxue Liu,*^1,2,*^ *Zhuoya Li,*^1^ *Xiaojia Guo,*^2^ *Xueying Wang,*^2^ *Zongbao K. Zhao*^2^

^1^ Henan Engineering Laboratory for Bioconversion Technology of Functional Microbes, College of Life Science, Henan Normal University, Xinxiang 453007, China

^2^ Laboratory of Biotechnology, Dalian Institute of Chemical Physics, Chinese Academy of Sciences, Dalian 116023, China

*Corresponding author.

**Supplementary information**:

1. Kinetic parameters of enzymes (Table S1)

2. Strains (Table S2)

3. Plasmids (Table S3)

4. Method used to determine the activity of NadD variants by detecting the transformation efficiency

5. Western blot assay (Figure S1)

6. Schematic presentation of constructing plasmids (Figure S2)

7. Uncropped immunoblot corresponding to supplementary Figure S1 (Figure S3).

**1. Kinetic parameters of enzymes**

Kinetic parameters of a series of previously enzymes used in this work are summarized in Table S1.

Table 1. Kinetic parameters of a series of previously enzymes

| Enzyme | Substrate | Kinetic parameters | | | Reference |
| --- | --- | --- | --- | --- | --- |
|  |  | *k*_cat_ (s^-1^) | *K*_m_ (mM) | *k*_cat_/*K*_m_ (mM^-1^ s^-1^) |  |
| Pdh | NAD | 0.24 ± 0.01 | 1.1 ± 0.1 | 218 | [1] |
|  | NCD | 0.28 ± 0.02 | 22.2 ± 3.9 | 12.6 |  |
| Pdh_I151R | NAD | 0.18 ± 0.01 | 78.8 ± 6.5 | 2.3 |  |
|  | NCD | 0.11 ± 0.02 | 15.4 ± 2.5 | 7.2 |  |
| Pdh_I151R/P176E | NAD | 0.31 ± 0.01 | 684.6 ± 35.5 | 0.45 |  |
|  | NCD | 0.06 ± 0.00 | 7.9 ± 0.3 | 6.96 |  |
| Pdh* | NAD | 0.2 1± 0.07 | 4.7×10^3^ ± 1.2×10^3^ | 0.045 |  |
|  | NCD | 0.20 ± 0.02 | 99.1 ± 14.8 | 2.04 |  |
| ME | NAD | 57.3 ± 0.4 | 0.27 ± 0.01 | 214.8 | [2] |
|  | NCD | 26.1 ± 4.1 | 9.4 ± 1.9 | 2.8 |  |
| ME* | NAD | 3.8 ± 0.4 | 10.4 ± 1.2 | 0.36 |  |
|  | NCD | 158.2 ± 17.8 | 1.02 ± 0.17 | 154.6 |  |
| NadD | ATP | 4.36 | 2.67×10^-2^ | 162 | [3] |
|  | CTP | 0.017 | 6.07 | 2.83×10^-3^ |  |
| NcdS-2 | ATP | 0.036 | 4.2×10^-2^ | 0.87 |  |
|  | CTP | 0.296 | 7.5×10^-3^ | 39.4 |  |
| NcdS-3 | ATP | 4.12 | 3.39×10^-2^ | 1.21 |  |
|  | CTP | 0.97 | 2.68×10^-2^ | 36.2 |  |

**2. Strains**

The strains used in this study are summarized in Table S2.

Table S2 Strains used in this study.

| Strains and Plasmids | Genotypes | Source |
| --- | --- | --- |
| BW14329 | *F-, Δ(argF-lac)169, ΔphoA532, λ-, rpoS396(Am), rph-1, Δ(phnP-mel)522(Tn5-1/132), hsdR514, creC510, Mu+* | CGSC |
| BW16787 | *F-*, *Δ(argF-lac)169*, *ΔphoA532*, *λ^-^*, *rpoS396*(*Am*), *rph-1*, *Δ(phnP-phnH)523(Tn5-1/132)*, *hsdR514*, *creC510*, *Mu+* | CGSC |
| BW16847 | *F-*, *Δ(codB-lacI)3*, *ΔphoA532*, *pdxH15*(*Am*), *purR106*::*Tn10*, *Δ(phnP-phnD)3330(phnC?)* | CGSC |
| BW22246 | *F-*, *Δ(codB-lacI)3*, *ΔphoA532*, *ΔuidA3*::*pir^+^*, *recA1*, *Δ(phnP-phnD)3330(phnC?)* | CGSC |
| BW25141 | *F-, Δ(araD-araB)567, ΔlacZ4787(::rrnB-3), Δ(phoB-phoR)580, λ-, galU95, ΔuidA3::pir+, recA1, endA9(del-ins)::FRT, rph-1, Δ(rhaD-rhaB)568, hsdR514* | CGSC |
| BW14329-YX00 | BW14329, pK | This study |
| BW14329-YX01 | BW14329, pK-Pdh | This study |
| BW14329-YX09 | BW14329, pK-Pdh_I151R | This study |
| BW14329-YX10 | BW14329, pK-Pdh* | This study |
| BW14329-YX11 | BW14329, pK-Pdh_I151R/P176E | This study |
| BW-PB01 | BW14329, pUC-chl-NcdS2+Pdh*, pTrc99K-ME* | This study |
| BW-PB03 | BW14329, pUC-chl-NcdS3+Pdh*, pTrc99K-ME* | This study |
| BW-PB05 | BW14329, pUC-chl-3G8+Pdh*, pTrc99K-ME* | This study |
| BW-PB07 | BW14329, pUC-chl-NcdS2+Pdh*, pTrc99K-ME | This study |

**3. Plasmids**

The plasmids used in this study are summarized in Table S3.

Table S3 Plasmids used in this study.

| Plasmids | Genotypes | Source |
| --- | --- | --- |
| pUC18 | *lacZ, pBR322 ori, bla*, cloning vector | Takara |
| pK | pUC18, *bla::kan* | [4] |
| pK-Pdh | pK with lacZ substituted by wild-type Pdh | [1] |
| pK-Pdh* | pK with lacZ substituted by Pdh* | [1] |
| pK-Pdh_I151R | pK with lacZ substituted by Pdh_I151R | [1] |
| pK-Pdh_I151R/P176E | pK with lacZ substituted by Pdh_I151R/P176E | [1] |
| pTrc99K-ME | pTrc99a with wild-type ME expression, *bla::kan* | Lab collection |
| pTrc99K-ME* | pTrc99a with ME-L310R/Q401C expression, *bla::kan* | Lab collection |
| pUC-kan-NadD | pK with wild-type NadD expression | [5] |
| pUC-kan-NadD* | pK with NadD variants expression, including 22C8, 23F7, 109H9, 1C1, and 22D8 | [5] |
| pUC-kan-3G8 | pK with NadD-V23Q/W176E expression | [3] |
| pUC-kan-NcdS2 | pK with NcdS2 expression | [3] |
| pUC-kan-NcdS3 | pK with NcdS3 expression | [3] |
| pUC-chl-(*P_araB_*)ME*+Pdh* | pUC with lacZ substituted by ME* with *ara* operon and Pdh* with *lac* operon, *bla::cat* | This study |
| pUC-chl-NcdS2+Pdh* | pUC with lacZ substituted by NcdS2 and Pdh* with *lac* operon, *bla::cat* | This study |
| pUC-chl-NcdS3+Pdh* | pUC with lacZ substituted by NcdS and Pdh* with *lac* operon, *bla::cat* | This study |
| pUC-chl-3G8+Pdh* | pUC with lacZ substituted by NadD-V23Q/W176E and Pdh* with *lac* operon, *bla::cat* | This study |

**4. Method of determining the activity of NadD variants by detecting the transformation efficiency**

Add 1 µg pUC-kan-NadD or pUC-kan-NadD* plasmid DNA and 1 µg pUC-chl-(*P*_araB_)ME*+Pdh* plasmid DNA within 100 µl electro-competent cells of BW14329, and mix by gentle pipetting. The cell-DNA mixture was transferred into an ice chilled 2 mm gap electroporation cuvette, and electroporated at 2.5kV. Immediately add 1 mL LB and and transfer into a microcentrifuge tube. Rescue at 37 °C with shaking for 45 min. Cells were collected by centrifugation at 10000×*g* at 4 °C for 2 min, washed thrice and resuspended with 1 mL of MOPS medium without P source. A 100 μL volume of cell suspension was coated on the MOPS agar plate with 0.4% glucose, 50 μg/mL kanamycin, 30 μg/mL chloramphenicol, 0.1 mM IPTG, 1 mM L-arabinose, and with 50 mM phosphite as the sole P source. After incubation at 25 °C for ~2 days, colonies formed were counted.

**5. Schematic presentation of constructing plasmids**


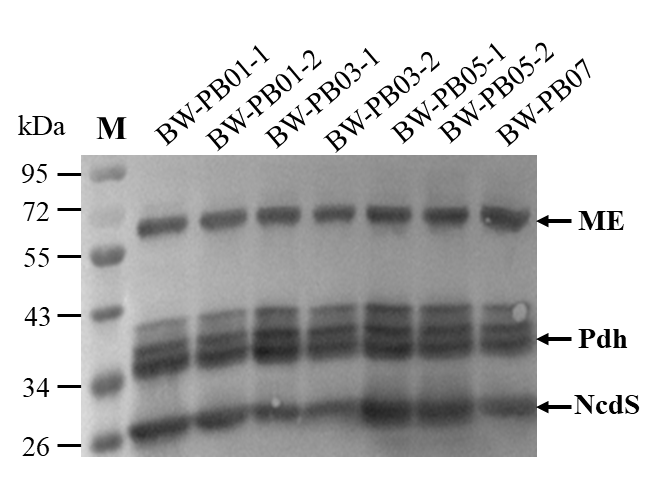


Figure S1 Expression of enzyme for engineered *E. coli* cultured in liquid minimal media with 5 mM Phi.


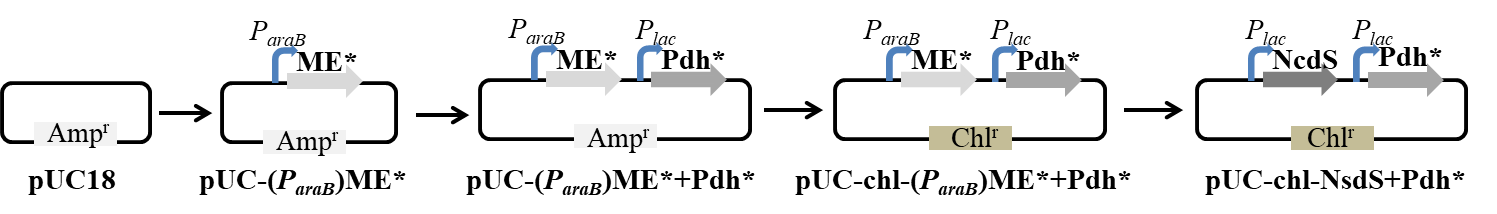


Figure S2 Schematic presentation of constructing plasmids. RF cloning strategy was used to construct the vectors.


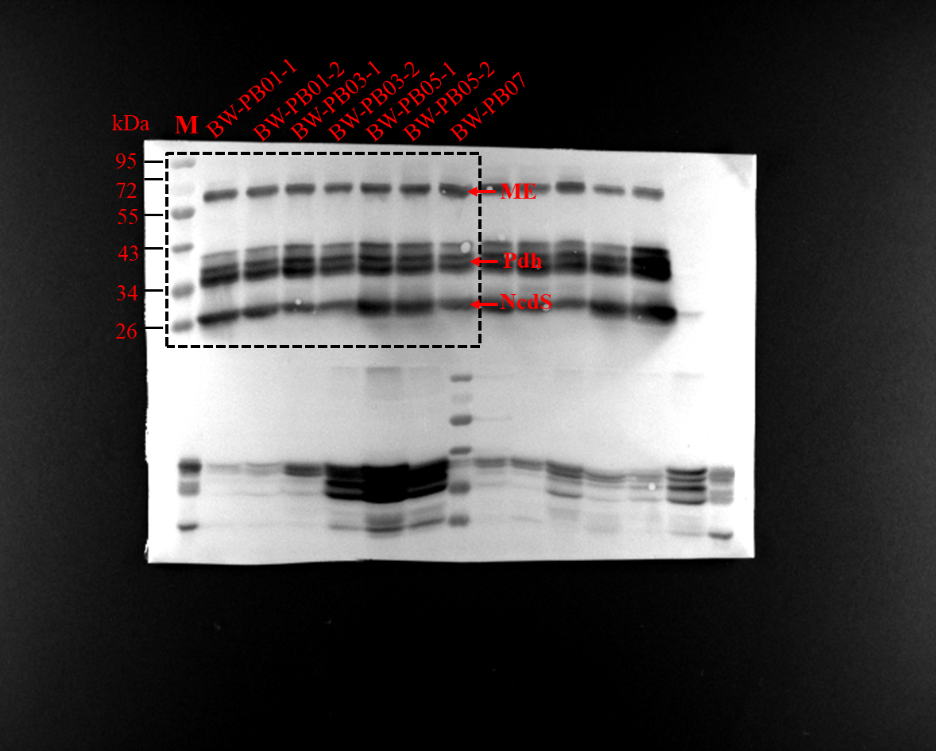


Figure S3 Uncropped immunoblot corresponding to Figure S1

**References**

1. Liu Y, Feng Y, Wang L, Guo X, Liu W, Li Q, Wang X, Xue S and Zhao ZK (2019) Structural insights into phosphite dehydrogenase variants favoring a non-natural redox cofactor. ACS Catal. 9:1883-1887. doi: 10.1021/acscatal.8b04822

2. Ji D, Wang L, Hou S, Liu W, Wang J, Wang Q and Zhao ZK (2011) Creation of bioorthogonal redox systems depending on nicotinamide flucytosine dinucleotide. J. Am. Chem. Soc. 133:20857-20862. doi: 10.1021/ja2074032

3. Wang X, Feng Y, Guo X, Wang Q, Ning S, Li Q, Wang J, Wang L and Zhao ZK (2021) Creating enzymes and self-sufficient cells for biosynthesis of the non-natural cofactor nicotinamide cytosine dinucleotide. Nat. Commun. 12:2116. doi: 10.1038/s41467-021-22357-z

4. Wang L, Ji D, Liu Y, Wang Q, Wang X, Zhou YJ, Zhang Y, Liu W and Zhao ZK (2017) Synthetic cofactor-linked metabolic circuits for selective energy transfer. ACS Catal. 7:1977-1983. doi: 10.1021/acscatal.6b03579

5. Wang X, Zhou YJ, Wang L, Liu W, Liu Y, Peng C and Zhao ZK (2017) Engineering *Escherichia coli* nicotinic acid mononucleotide adenylyltransferase for fully active amidated NAD biosynthesis. Appl. Environ. Microbiol. 83:e00692-17. doi: 10.1128/AEM.00692-17
